# Long-Term Intravenous Ketamine for Analgesia in a Child with Severe Chronic Intestinal Graft versus Host Disease

**DOI:** 10.1155/2015/834168

**Published:** 2015-12-08

**Authors:** Jennifer Busse, Leroy Phillips, William Schechter

**Affiliations:** ^1^Anesthesiology, Morgan Stanley Children's Hospital at Columbia University, New York, NY 10032, USA; ^2^Anesthesiology, New York University Langone Medical Center, New York, NY 10016, USA; ^3^Anesthesiology and Pediatrics, Morgan Stanley Children's Hospital at Columbia University, New York, NY 10032, USA

## Abstract

Ketamine is reported to be an effective adjuvant to opioids in the treatment of refractory cancer pain; however, the use of high doses of ketamine for extended periods in pediatric patients has not been described. We present a five-year-old male with grade IV intestinal GVHD whose abdominal pain required both hydromorphone and ketamine for a period of over four months. There was no evidence of hepatotoxicity, hemorrhagic cystitis, or other adverse effects. Possible withdrawal symptoms were mild and were readily mitigated by gradually weaning ketamine.

## 1. Introduction

Chronic pain related to gastrointestinal graft versus host disease (GVHD) is often difficult to treat, causing a significant decrease in quality of life [1]. The pain is characterized as sharp or cramping and is attributed to inflammation, edema, increased mucosal friability, and ileus [2]. GVHD symptoms present as anorexia, nausea, vomiting, diarrhea, and cholestasis and may be accompanied by a maculopapular rash. Opioids are commonly utilized to treat the chronic pain related to GVHD, but their efficacy is limited by associated adverse effects, including sedation [3], immunosuppression [4], nausea, vomiting, and ileus. Nonsteroidal anti-inflammatories (NSAIDs) or acetaminophen may be contraindicated because of hepatic insufficiency or coagulopathy.

Ketamine has been reported to be effective as an adjuvant to opioids in the treatment of chronic refractory cancer pain [5–7]. It is an N-methyl-D-aspartate (NMDA) receptor antagonist which induces synthesis and release of nitric oxide and indirectly modulates mu opioid receptor signaling [8, 9]. In chronic pain, ketamine reduces central sensitization [10] and decreases the development of opioid tolerance [11]. Doses of 0.2 mg/kg/h to 0.5 mg/kg/h of IV ketamine given for short periods have been shown to be safe and opioid-sparing in pediatric patients [12]. Studies in adults have shown long-term control of cancer-related pain via the oral [13–16], intravenous (IV), or subcutaneous route [16–19]. Common adverse effects associated with ketamine include blurred vision, sialorrhea, anorexia, depression, hypertension, insomnia, anxiety, and visual and dream disturbances [19–21]. Long-term administration of ketamine may be associated with transaminitis and ulcerative or hemorrhagic cystitis [22, 23]. Its use is contraindicated in patients with increased intracranial or intraocular pressure.

There have been few studies on the use of ketamine in pediatric patients and only one prospective study of long-term ketamine use for chronic pain in pediatric patients via oral administration [12, 13]. The only case study of long-term administration of intravenous ketamine in pediatric patients was over a period of nine weeks to treat cancer pain. The dose administered was not reported, and side effects observed included myoclonic movements [24]. Previous case studies among adults who chronically misused ketamine for many months have variously reported withdrawal symptoms [25, 26]. Abrupt ketamine discontinuation has been shown to increase allodynia, hyperalgesia, sleep disorders, and anxiety in adults [27–29]. Higher dose ketamine for treatment of pain in pediatric patients over an extended time period has not been reported. We now report the use of higher dose ketamine infusion for a prolonged period to treat pain associated with GVHD in a 5-year-old child without evidence of toxicity, tolerance, or signs of withdrawal during infusion.

## 2. Case Description

A five-year-old 27.5 kilogram male with history of high-risk acute myeloid leukemia received a matched unrelated donor transplant at an outside institution. His course was complicated by persistent vancomycin resistant* Enterococcus* infection and grade IV chronic intestinal GVHD refractory to multiple therapies. He was transferred to our institution specifically for treatment of GVHD using a mesenchymal stem cell therapy (MSC) protocol [30, 31]. Epidural infusion of a local anesthetic and an opioid initially provided excellent control of abdominal pain. Because of persistent fevers, the epidural was discontinued and the patient was treated with hydromorphone utilizing patient controlled analgesia (PCA) but required the subsequent addition of a ketamine infusion that was incrementally titrated to higher doses. Just prior to transfer to our institution, his abdominal pain was well controlled on 28 mg per day of hydromorphone and 0.54 mg/kg/h of ketamine which had been slowly titrated up by an outside hospital over four months. The patient remained on ketamine at our institution for an additional 6 weeks.

Abdominal pain was described as constant, of severe intensity, and cramping extending from the suprapubic area to the umbilicus. When ketamine was significantly reduced to 0.054 mg/kg/h (0.9 mcg/kg/min) after his transfer, pain scores increased significantly. When raised back to 0.54 mg/kg/h (9 mcg/kg/min), pain control was regained. On exam, bowel sounds were hyperactive and the abdomen was tender and distended but without rebound tenderness. There was mild hepatomegaly, but no evidence of free or intramural intestinal air. Mental status was normal and he was not overly sedated. There was no diplopia, sialorrhea, or hematuria. Despite treatment, he had persistent vomiting and unremitting diarrhea. Blood pressure was elevated upon admission and remained so with an average of 120/80, which is approximately the 99th percentile for his age. Hypertension was thought to be related to tacrolimus and/or steroids. Hypertension was treated with a clonidine patch, carvedilol, amlodipine, furosemide, and enalapril.

Upon admission, a CT scan of the abdomen and pelvis showed bowel wall thickening. Liver function testing was closely monitored during ketamine treatment both for toxicity and for signs of venoocclusive disease or GVHD of the liver. Alanine aminotransferase never exceeded normal values; however, aspartate aminotransferase was mildly elevated but stable from before his transfer. Total, direct, and indirect bilirubin were also mildly elevated, but never to more than 10% of the upper limit of normal. Alkaline phosphatase and lactate dehydrogenase were both persistently elevated averaging 275 U/L and 580 g/dL. Liver function enzymes returned to normal levels within one week of discontinuation of ketamine and completion of the MSC protocol.

Clinical improvement in GVHD was noted with MSC therapy. Opioid dosing via PCA was weaned by approximately 20% of the total daily dose every 48 hours. On alternate days, the ketamine infusion dose was reduced by 1 mcg/kg/minute with no recrudescence of abdominal pain. Mild irritability and restlessness were noted as the opioid and ketamine were weaned. These symptoms resolved within 3 days of discontinuance of ketamine and IV opioids. It was not clear whether this represented a distinct ketamine withdrawal phenomenon; however, there was no increase in the number of bowel movements, nausea, or tremors to suggest opioid withdrawal. A clonidine patch remained in place throughout the weaning process.

## 3. Discussion

Pain related to gastrointestinal GVHD can be persistent and difficult to treat. Ketamine is an excellent adjuvant for the management of chronic cancer pain [3, 12–18], but never specifically as a treatment for pain associated with GVHD in pediatrics. Safe dose limits, duration of treatment, or a withdrawal syndrome have not been established in pediatrics. In this case, ketamine was an excellent adjuvant to hydromorphone, providing a clinically observable improvement in pain scores and a plateau in total opioid requirement. This may be related to the additional analgesia provided by ketamine, as well as ketamine's mitigating effects on the development of opioid tolerance. Ketamine may have also prevented the development of gastroenteric nerve sensitization and hyperalgesia, due to inhibition of inflammatory cytokines known to accompany GVHD [31].

NSAIDs and acetaminophen were contraindicated because of risk of bleeding and hepatotoxicity risks. Tricyclic antidepressants, SSRIs, and anticonvulsants could not be used because the enteral route was not reliably available. A ketamine infusion was selected over other pharmacologic pain treatment modalities including a lidocaine infusion because the risk of an adverse event was thought to be less.

Ketamine infused at higher doses for longer periods of time than previously reported appeared to be safe in this patient. Our case also shows that tolerance to ketamine may develop over time. Theoretically, a mild withdrawal syndrome may have been mitigated to some extent by the use of clonidine, an alpha 2 agonist. Clonidine modulates the presynaptic release of norepinephrine [32]; therefore, one might speculate that its presence might affect ketamine withdrawal, since ketamine is known to increase endogenous norepinephrine release [33]. Resolution of abnormal liver function occurred after discontinuation of ketamine. However, since the patient had abnormal liver function even before the initiation of ketamine administration, it is unlikely that this was related to the use of ketamine. Our case illustrates that long-term administration of ketamine may be associated with very little significant adverse effects in some patients. Therefore, long-term administration of ketamine appeared safe in this case and may be considered in severe, refractory pain syndromes associated with GVHD but may require careful uptitration over time. It is important to emphasize that the patient should be closely monitored for excessive sedation, liver toxicity, hematuria, hypertension, tachycardia, or psychomimetic effects. Slow weaning is recommended to prevent the manifestation of withdrawal symptoms.
